# Influence of Staging and Grading and Multiple Factors on the Success of Non‐Surgical Periodontal Therapy Performed by Dental Hygienists: A Retrospective Analysis

**DOI:** 10.1111/idh.70036

**Published:** 2026-02-22

**Authors:** Matteo Serroni, Obada Mandil, Chiara Galano, Aml Abukraa, Aliyah Alsubhi, Danielle Rulli, Muhammad H. A. Saleh, Andrea Ravidà

**Affiliations:** ^1^ Department of Periodontics and Preventive Dentistry, School of Dental Medicine University of Pittsburgh Pittsburgh Pennsylvania USA; ^2^ Department of Innovative Technologies in Medicine & Dentistry University ‘G. D'Annunzio’ of Chieti‐Pescara Chieti Italy; ^3^ Department of Periodontics and Oral Medicine, School of Dentistry University of Michigan Ann Arbor Michigan USA; ^4^ Department of Periodontics Case Western Reserve University School of Dental Medicine Cleveland Ohio USA; ^5^ Private Practice Napoli Italy; ^6^ Herman Ostrow School of Dentistry of USC Los Angeles California USA; ^7^ Division of Dental Hygiene Ohio State University College of Dentistry Columbus Ohio USA

## Abstract

**Objective:**

To evaluate the incomplete success rate (ISR) of non‐surgical periodontal therapy (NSPT) performed by registered dental hygienists, considering periodontitis staging and grading and multiple tooth‐ and patient‐level factors.

**Methods:**

Data from patients with periodontitis treated by registered dental hygienists at the University of Michigan were analysed. A complete medical history, periodontal chart and full‐mouth radiographs were collected before NSPT (T0), and clinical periodontal parameters were recorded at re‐evaluation (T1). Tooth‐level ISR (tISR) was defined as the percentage of teeth with residual pocket depth (PD) > 4 mm [Condition 1 (C1)], or PD of 5 mm with bleeding on probing, or PD ≥ 6 mm [Condition 2 (C2)]. Patient‐level ISR (pISR) was calculated as the percentage of patients with at least one tooth meeting the criteria for C1 or C2 at T1. Multivariate analyses assessed the impact of staging, grading and other tooth‐ and patient‐level factors on ISR outcomes following NSPT.

**Results:**

A total of 1818 teeth from 133 patients were re‐evaluated 113.2 ± 85 days after NSPT. The tISR was 28.6% and 20.6% for C1 and C2, respectively; while the pISR was 75.9% and 65.4%. Factors linked to higher tISR for both C1 and C2 included baseline PD and multi‐rooted teeth. Smokers and former smokers had more teeth with pathological sites under C1. Higher tISR under C2 was observed in cases presenting radiographic bone loss > 33%. Advanced stage of periodontitis significantly predicted higher pISR for C1, with Stage III‐IV patients over twice as likely to exhibit higher pISR than Stage II (OR = 2.35; *p* = 0.042).

**Conclusion:**

Advanced periodontitis stage at T0 significantly predicts higher pISR values, particularly for C1. tISR after NSPT is influenced by baseline PD, number of roots for both conditions and smoking for C1.

## 1 Introduction

The main objective of periodontal therapy is to prevent the further progression of periodontitis by avoiding the consequent loss of the natural dentition [1]. The key role of prevention and treatment of periodontitis should be emphasised, considering all the negative implications of the disease, including increased risk of systemic conditions (e.g., diabetes, cardiovascular diseases, etc.) [2, 3]. The 2020 guidelines of the European Federation of Periodontology (EFP) have described a pre‐established stepwise approach for treating periodontitis of various Stages. The first step in the treatment is aimed at controlling risk factors (systemic and local), professionally removing supragingival biofilm and calculus, and guiding behavioural change by motivating the patient to implement oral hygiene procedures effectively. Second, the therapeutic approach is aimed at controlling/eliminating subgingival biofilm and calculus, and after the resulting healing period, the patient's eligibility for any additional therapies in the third step is assessed [4].

There is certain heterogeneity in the literature in defining the success criteria as well as the critical pocket depth, which unequivocally indicates surgical therapy, or which can determine a greater risk of tooth loss or progression of the disease [5, 6, 7]. In general, the presence of shallow PD free from bleeding on probing (BoP) represents the main indicator of the success of periodontal therapy [8]. A review by Graziani et al. concluded by arguing that the aim of periodontal treatment should be the ‘closure’/elimination of pockets ≥ 5mm [9]. In fact, residual PD ≥ 5 mm was considered a site‐specific positive predictive factor for further clinical attachment loss during supportive periodontal therapy (SPT), and numerous authors used a PD ≤ 4 mm as an endpoint to define the pocket ‘closure’ [5, 10, 11]. A retrospective 11‐year study concluded that a residual pocket with a PD of 5 mm posed a risk factor for tooth loss, with an odds ratio of 7.7 compared to pockets with a PD of ≤ 3 mm [12].

Currently, the latest EFP guidelines define the endpoints of therapy as the absence of periodontal pockets > 4 mm with bleeding on probing (BoP) or deep pockets (≥ 6 mm), the persistence of which would indicate the need for the implementation of step three of periodontal therapy [4].

The staging and grading system introduced in the 2018 classification of periodontal diseases aims to consistently define the current severity of periodontitis, its impact on the required treatment, and guide clinicians in identifying factors that influence the risk of disease progression and appropriate management strategies [13]. Recent studies have explored the prognostic potential inherent in the 2018 classification, highlighting that patients classified in more advanced stages and grades exhibit significantly higher rates of periodontitis‐related tooth loss [14, 15]. Conversely, other research has sought to determine whether the 2018 classification system could predict treatment response but has been unable to identify specific aspects of staging and grading that correlate with the response pattern to non‐surgical periodontal therapy (NSPT) [16].

Currently, there is limited evidence that has analysed the impact of disease severity, as defined by the staging and grading in the 2018 AAP/EFP Classification, on the success of periodontal therapy [3]. The aim of the present study was to investigate the incomplete success rate of NSPT exclusively performed by dental hygienists, identifying both the patients and teeth potentially requiring intervention by a periodontist. Therapeutic outcomes were primarily evaluated based on the stage and grade of periodontitis, while also considering a range of systemic, local, clinical and radiographic factors.

## 2 Materials and Methods

This retrospective cohort study was conducted in accordance with the 1975 Declaration of Helsinki, as most recently revised in 2013. Approval by the University of Michigan, School of Dentistry, Institutional Review Board for Human Studies (IRBMED) was obtained with the study identifier HUM00215936.

The manuscript was produced following the STROBE (Strengthening the Reporting of Observational Studies in Epidemiology) guidelines. All data in the present study were retrospectively collected from the records of patients who were screened and treated by faculty and staff‐registered dental hygienists between August 2013 and September 2021 at the University of Michigan School of Dentistry, Ann Arbor, MI.

*Inclusion criteria*:

- Patients meeting the case definition of periodontitis as defined by the 2017 world workshop [3].
- Patients treated exclusively by faculty or staff registered dental hygienists for periodontal disease at the University of Michigan School of Dentistry.
- Patients who underwent at least one session of SRP and received only a single cycle of instrumentation without any additional therapy, whether local (e.g., physical or chemical agents), systemic (e.g., systemic antimicrobials), or with host‐modulating agents (local or systemic).
- Patients with a complete periodontal record and at the first follow‐up (revaluation) after non‐surgical periodontal therapy (T1).
- Patients with full‐mouth radiographs available at baseline, taken prior to the initiation of active therapy (T0).

*Exclusion criteria*:

- Patients with files inaccessible due to bad credit, documents destroyed or deceased.
- Patients with incomplete data for conducting the Staging and Grading properly.
- Patients where initial (T0) and re‐evaluation (T1) periodontal charts were performed by different hygienists.
- Patients who have not performed the SRP session completely with hygienists.
- Patients are already undergoing periodontal therapy within the last 2 years.

### 2.1 Data Collection and Patient Classification

Physical and digital data extracts from the electronic records of patients who met the predetermined eligibility criteria were evaluated and collected by two examiners (OM and AA). First, general information (e.g., age and gender) and relevant aspects of each patient's medical history (e.g., history of smoking or diabetes) were reported.

The PD and clinical attachment level (CAL) values were recorded at six sites for each tooth, but only the highest PD reading, along with its corresponding CAL value, was assigned to each tooth during data transfer.

The PD was measured as the distance in millimetres between the bottom of the pocket and the free gingival margin. The CAL was evaluated in mm as the distance between the bottom of the pocket and the cementum‐enamel junction.

A single value was also reported relating to the worst degree of furcation involvement for multi‐rooted teeth, as well as the presence (yes/no) of vertical bone defects (with infrabony component > 3 mm) for each element. BoP was also recorded. The presence of BoP (yes/no) was attributed to the site that, at baseline and T1, was recorded as having the highest PD value.

Calculation of the level of radiographic bone loss (RBL, %) was performed primarily by examining periapical radiographs taken within 6 months of initial treatment. Measurements were performed using a calibration tool overlayed between the CEJ at the root apex of each individual tooth. The instrument is shaped like a ruler with three coloured bands: green for RBL < 15%, yellow for RBL 15%–33% and red for RBL > 33% [17]. The presence of restorations on interproximal surfaces and prosthetic crowns at T0 and T1 was collected.

Before staging and classification were established, the patient had to meet the 2018 World Workshop case definition of periodontitis [3].

The staging and grading algorithms outlined by Tonetti and Sanz were employed to categorise patients based on their stage (I‐IV) and grade (A‐C), using fundamental clinical and radiographic parameters recorded at T0 [18]. Extent was calculated after determining the stage and evaluated as the percentage of teeth at the severity level defining the stage [19]. The staging and classification process were independently carried out by two researchers (MS and OM) using clinical data collected prior to initiating initial active periodontal therapy (T0). In instances of uncertainty or disagreement in assigning a participant to a specific stage and/or grade, the case was reviewed by a third examiner (AR) until a consensus was reached.

### 2.2 Outcomes Measures

The primary outcome of the study was the ‘incomplete success rate’ (ISR) achieved by the hygienist following non‐surgical periodontal therapy, assessed at both the patient and tooth levels during the periodontal re‐evaluation conducted at T1. To calculate both the tooth‐level IRR (tIRR) and patient‐level IRR (pIRR), two distinct conditions were evaluated separately. Specifically, the tIRR was defined as the percentage of teeth at T1 that exhibited persistence at the analysed site based on the following two conditions: (1) PD > 4 mm [Condition 1 (C1)], or (2) PD = 5 mm with BoP+ or PD > 6 mm [Condition 2 (C2)].

Similarly, the patient‐level IRR (pIRR) was defined as the percentage of patients with at least one tooth meeting the criteria for tIRR, considering both C1 and C2.

### 2.3 Data Analysis

The primary endpoint of the study was the ISR assessed at both the tooth level (tISR) and the patient level (pISR) after non‐surgical periodontal therapy at T1, taking into account both C1 and C2.

At the tooth‐level, for both conditions, the outcome tISR was related to all independent variables using multi‐level binary logistic regression with generalised estimation equations (GEE) on the sample of teeth with pathological PD (≥ 4 mm) at baseline. Raw odds ratios and 95% confidence intervals were obtained from Wald's Chi [2] statistical analysis. Then, multiple models were estimated to adjust for potential confounding factors. The reliability of fit of different estimations (for different matrix correlations) was assessed by QIC statistics.

At the patient‐level, similar models were conducted (binary logistic regression) to analyse the outcome pISR obtained by the hygienist. Raw odds ratios and 95% confidence intervals were obtained.

The significance level used in analysis has been 5% (*α* = 0.05). Regarding the power analysis, a post hoc estimation was obtained. A sample size of 1818 independent teeth with PD ≥ 4 mm provides 99.9% power at confidence 95% to detect a complete success rate of 2% and 10% as significant using a logistic regression model. However, teeth were not independent, and this power must be corrected because of the two‐level structure of data. Each patient provided an average of 13.7 teeth, and within‐subject correlation CCI = 0.5 (moderate) was assumed, leading to a correcting coefficient *D* = 7.33. Therefore, 1818 dependent teeth provide the same power as 248 independent teeth, estimated at 70.7% under the same previous conditions.

Additional analyses were performed using the receiver operating characteristic (ROC) curve to estimate the maximum PD threshold value at T0 capable of predicting the tISR by the hygienist, considering both C1 and C2. The PD threshold value was determined at the point where the sum of the diagnostic validity indices, sensitivity (SEN) and specificity (SPE), represented by the Youden index, reached its maximum. The area under the ROC curve (AUC) was used to evaluate diagnostic accuracy.

## 3 Results

### 3.1 Demographic Results

From an initial electronic database of 178 patients, a total of 133 patients (61 females and 72 males) were included in the study, with a mean age at intake of 52.8 ± 15.4 years (range 21–88) and a total of 2249 teeth, of which 1818 had at least one pathological site with PD ≥ 4 mm. All eligible screened patients underwent non‐surgical periodontal therapy in a time interval between August 2013 and September 2021. All relevant descriptive tooth‐ and patient‐level characteristics are detailed in Table 1.

**TABLE 1:** Clinical and demographic characteristics of the study population at T0.

Characteristics tooth‐level	Total (*N* or mean ± SD)	Characteristics patient‐level	Total (*N* or mean ± SD)
Age (years)	49.6 ± 15.4	No. of patients	133
Total teeth with pathological sites (≥ 4 mm)	1818		
Sex		Age (years)	52.8 ± 15.4
Male	1053 (57.9%)	Sex	
Female	765 (42.1%)	Male	72 (54.1%)
Smoking		Female	61 (45.9%)
No	1214 (66.8%)	Smoking	
Former	272 (15.0%)	No	87 (65.4%)
Current	332 (18.3%)	Former	22 (16.5%)
Diabetes		Current	24 (18.0%)
No		Diabetes	
Yes	1578 (87.2%)	No	116 (87.9%)
Tooth type	232 (12.8%)	Yes	16 (12.1%)
I		Stage	
C	360 (19.8%)	2	62 (46.6%)
PM	226 (12.4%)	3–4	71 (53.4%)
M	569 (31.3%)	Grade	
Arch		A	31 (23.3%)
Maxilla	937 (51.5%)	B	73 (54.9%)
Mandible	881 (48.5%)	C	29 (21.8%)
Mean PD at T0	5.0 ± 1.0 mm	Grade in Stage II	
Teeth at T0 with sites of:	1382 (76%)		
4–5 mm	293 (16.15)		
6 mm	143 (7.9%)		
≥ 7 mm			
Mean CAL at T0	4.9 ± 1.4 mm	2A	29 (46.8%)
RBL at T0		2B	30 (48.4%)
< 15%	432 (24.1%)	2C	3 (4.8%)
15%–33%	1085 (60.5%)	Grade in Stage III	
> 33%	276 (15.4%)	3A	2 (2.8%)
VERTICAL DEFECT > 3 mm		3B	43 (60.6%)
No	1659 (92.5%)	3C	26 (36.6%)
Yes	135 (7.5%)	Extent	
BOP T0		Localised	55 (41.4%)
No	406 (23.3%)	Generalised	78 (58.6%)
Yes	1338 (76.7%)		
Prosthetic crown			
No	1614 (89.4%)		
Yes	191 (10.6%)		
Interprox. restoration			
No	1604 (88.8%)		
Yes	202 (11.2%)		

Abbreviations: BoP, bleeding on probing; C, canine; CAL, clinical attachment level; I, incisor; M, molar; PD, pocket depth; PM, premolar; RBL, radiographic bone loss; SD, standard deviation.

### 3.2 Categorisation of Patients According to the 2018 Classification

The entire cohort of 133 patients was divided according to the 2018 classification. 62/133 (46.6%) patients were classified as Stage II, of which 29/62 (46.8%) as Grade A, 30/62 (48.4%) as Grade B and 3/62 (4.8%) as Grade C. A total of 71/133 (53.4%) were diagnosed with Stage III periodontitis, of which 2/71 (2.8%) had Grade A, 43/71 (60.6%) with Grade B and 26/71 (36.6%) with Grade C. Periodontal disease was classified as localised in 55/133 patients (41.4%) and as generalised in 78/133 patients (58.6%).

### 3.3 Patient‐Level Results

All relevant descriptive patient‐level characteristics at baseline are detailed in Table 1.

#### 3.3.1 C1

In total, 24.1% of patients showed residual probing depths ≤ 4 mm at periodontal re‐evaluation (95% CI: 16.8%–31.3%) while 75.9% (101/133) of patients displayed at least one pocket of > 4 mm (95% CI: 68.7%–83.2%).

Based on the results of the univariate analysis, presented in Table S1a, it was observed that only the stage of periodontitis at baseline emerged as significant factors influencing the probability of showing incomplete pocket ‘closure’; diabetes showed a bordeline association with the outcome (*p* = 0.098). Therefore, a multiple model was estimated with diabetes and stage as potential predictors (Table 2a).

**TABLE 2:** (a) Incomplete success rate (pISR) assessed based on patient‐related clinical variables influencing the presence of C1. (b) Incomplete success rate (pISR) assessed based on patient‐related clinical variables influencing the presence of C2. Results of multiple binary logistic regression using GEE.

	Total	Incomplete success rate (pISR)	OR	95% CI	*p*
(a)					
No. of patients	133	101 (75.9%)			
Diabetes					
No	116 (87.9%)	84 (72.4%)	1		
Yes	16 (12.1%)	16 (100.0%)	4.76	0.59–38.2	0.142
Stage					
2	62 (46.6%)	42 (67.7%)	1		
3–4	71 (53.4%)	59 (83.1%)	2.35	1.03–5.37	0.042*
(b)					
No. of patients	133	87 (65.4)			
Diabetes					
No	116 (87.9)	72 (62.1)	1		
Yes	16 (12.1)	14 (87.5)	3.55	0.74–16.9	0.112
Stage					
2	62 (46.6)	33 (53.2)	1		
3–4	71 (53.4)	54 (76.1)	1.81	0.72–4.51	0.206
Grade					0.292
A	31 (23.3)	14 (45.2)	1		
B	73 (54.9)	51 (69.9)	2.13	0.79–5.74	0.136
C	29 (21.8)	22 (75.9)	2.51	0.65–9.70	0.183
C vs. B (ref.)			1.18	0.41–3.38	0.759

Abbreviations: CI, confidence interval; OR, odds ratio; pIRR, patient‐level incomplete resolution rate.

* *p* < 0.05, Wald test.

**FIGURE 1:**
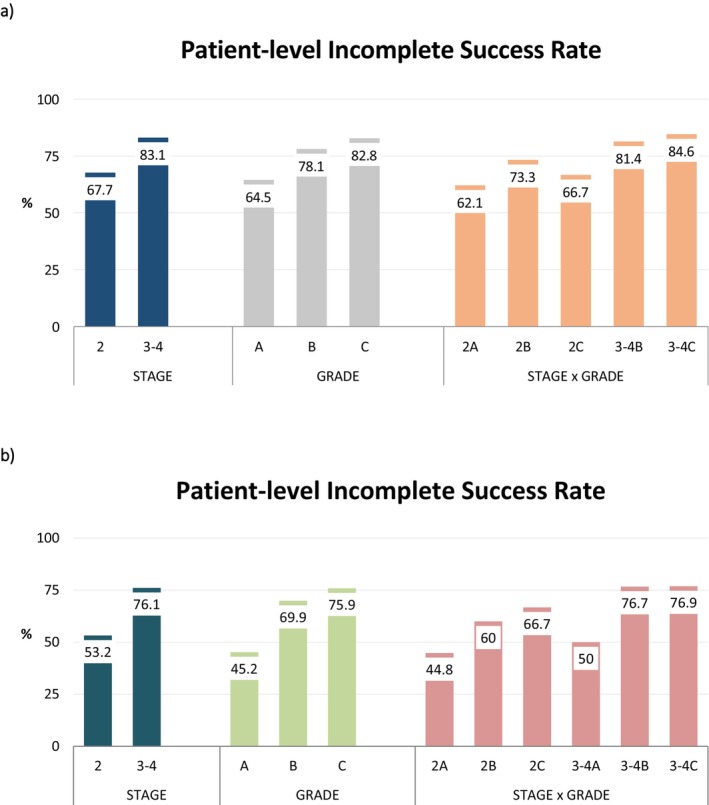
Patient‐level Incomplete Success Rate (pISR) according to Stage, Grade and interaction between Stage and Grade, considering incomplete success criteria as: (a) C1 and (b) C2.

Patients with Stage III‐IV presented 135% higher probability of incomplete pocket ‘closure’ (OR = 2.35, 95% CI: 1.03–5.37; *p* = 0.042) than patients with Stage II. The pISR relating to each Stage and Grade is shown in Figure 1a. Specifically, it was found that the percentage of patients in stages 3–4 who were free of teeth with pathological sites was significantly lower than that of patients in stages 2, with rates of 33.3% and 17.9%, respectively. No significant association (*p* = 0.142) emerged in the multiple models between diabetes and higher pISRs at T1.

A complementary analysis aimed at evaluating the risk of achieving incomplete success of periodontal therapy in Stage III‐IV patients, with or without vertical bone defects (with an infrabony component > 3 mm), found no statistically significant difference between the two groups in the multivariate model (*p* = 0.195) (Table S2a). Conversely, Stage III‐IV patients with baseline PD values of ≥ 7 mm showed a significantly higher probability (OR = 10.8, 95% Cl: 4.19–146.5; *p* < 0.001) of having at least one tooth with a residual PD > 4 mm compared to those with baseline PD ≤ 6 mm. The pIRR was 94.9% and 68.8%, respectively (Table S3a).

#### 3.3.2 C2

Overall, 34.6% (46/133) of patients meeting the C2 criteria demonstrated complete success of NSPT at the periodontal re‐evaluation (95% CI: 26.5%–42.7%). Conversely, 65.4% (87/133) presented a residual PD of 5 mm with BoP or ≥ 6 mm at T1 at the site with the greatest PD in at least one tooth (95% CI: 57.3%–73.5%).

Based on the results of the univariate analysis (Table S1b), it was observed that only the staging and grading of periodontitis at baseline emerged as significant factors influencing the likelihood of incomplete pocket ‘closure’. Diabetes demonstrated a trend toward significance (*p* = 0.062). Consequently, a multivariate model was developed using these predictors (Table 2b). However, no significant association between the variables considered and an increased risk of pISR was identified in the multivariate models. The percentage of patients in Stage 3–4 and Stage 2 who had no teeth with pathological sites was 23.9% and 46.8%, respectively (Figure 1b).

The multiple models of a sub‐analysis showed that Stage III‐IV patients with vertical bone defects (with an infrabony component > 3 mm) had a significantly higher risk of pISR compared to patients without infrabony defects at baseline (OR = 3.97, 95% CI: 1.04–15.1; *p* = 0.043) (Table S2b). Furthermore, Stage III–IV patients with teeth exhibiting higher baseline PD values of ≥ 7 mm had a significantly higher probability (OR = 24.8, 95% CI: 4.19–146.5; *p* < 0.001) of having at least one tooth with a C2 compared to those with baseline PD ≤ 6 mm. The pISR was 94.9% and 53.1%, respectively (Table S3b).

### 3.4 Tooth‐Level Results

The 1818 teeth with at least one pathological site (≥ 4 mm) had an overall mean PD at baseline of 5.0 ± 1.0 mm, considering their site with the highest PD. Of these, 1382 teeth (76%) had sites with PD between 4–5 mm, 293 teeth (16.15%) had sites with PD of 6 mm and 143 teeth (7.9%) had sites with PD ≥ 7 mm. All relevant descriptive tooth‐level characteristics at baseline are detailed in Table 1.

#### 3.4.1 C1

In total, 28.6% of the treated teeth reported PD values of > 4 mm at re‐evaluation (95% CI: 26.5%–30.7%) while 71.4% reported PD values of ≤ 4 mm (95% CI: 69.3%–73.5%).

Univariate analysis of the association between independent tooth‐related variables and tISR is displayed in Table S4a. Statistically significant variables were then analysed with multi‐level binary logistic regression with GEE (Table 3a).

**TABLE 3:** Results of multiple binary logistic regression using GEE on the sample of teeth with pathological PD (≥ 4 mm) at baseline (a) Incomplete success rate (tISR) assessed based on tooth‐related clinical variables influencing the presence of C1. (b) Incomplete success rate (tISR) assessed based on tooth‐related clinical variables influencing the presence of C2.

	Total	Incomplete success rate	OR	95% CI	*p*
(a)					
No. of teeth	1818	520 (28.6)			
Smoking					0.048*
No	1214 (66.8)	294 (24.2)	1		
Former	272 (15.0)	93 (34.2)	2.02	1.02–4.01	0.044*
Current	332 (18.3)	133 (40.1)	2.19	1.01–4.78	0.048*
Current vs. Former (ref.)			1.10	0.48–2.56	0.817
Diabetes					
No	1578 (87.2)	417 (26.4)	1		
Yes	232 (12.8)	100 (43.1)	1.11	0.50–2.48	0.802
Molar					
No	1155 (63.5)	219 (19.0)	1		
Yes	663 (36.5)	301 (45.4)	1.43	0.82–2.51	0.208
Number of roots					
Single	1019 (56.1)	181 (17.8)	1		
Multi	799 (43.9)	339 (42.4)	2.30	1.37–3.88	0.002**
PD at T0					< 0.001***
4–5 mm	1382 (76.0)	221 (16.0)	1		
6 mm	293 (16.1)	174 (59.4)	5.59	3.60–8.69	< 0.001***
≥ 7 mm	143 (7.9)	125 (87.4)	16.9	6.01–47.7	< 0.001***
≥ 7 mm vs. 6 mm (ref.)			3.14	1.07–9.17	0.036*
Mean CAL at T0	4.9 ± 1.4		1.10	0.94–1.27	0.234
RBL at T0					0.223
< 15%	432 (24.1)	87 (20.1)	1		
15%–33%	1085 (60.5)	295 (27.2)	1.16	0.68–1.97	0.586
> 33%	276 (15.4)	137 (49.6)	1.89	0.91–3.93	0.090
> 33% vs. 15%–33% (ref.)			1.63	0.87–3.03	0.130
Vertical defect > 3 mm					
No	1659 (92.5)	451 (27.2)	1		
Yes	135 (7.5)	69 (51.1)	1.56	0.85–2.86	0.152
BOP at T0					
No	406 (23.3)	87 (21.4)	1		
Yes	1338 (76.7)	413 (30.9)	1.62	0.91–2.88	0.099
(b)					
No. of teeth	1818	375 (20.6)			
Smoking					0.196
No	1214 (66.8)	217 (17.9)	1		
Former	272 (15.0)	65 (23.9)	1.59	0.69–3.70	0.278
Current	332 (18.3)	93 (28.0)	1.96	0.90–4.27	0.090
Current vs. former (ref.)			1.23	0.47–3.22	0.672
Diabetes					
No	1578 (87.2)	296 (18.8)	1		
Yes	232 (12.8)	77 (33.2)	1.00	0.40–2.50	0.997
Molar					
No	1155 (63.5)	154 (13.3)	1		
Yes	663 (36.5)	221 (33.3)	1.22	0.65–2.28	0.540
Number of roots					
Single	1019 (56.1)	126 (12.4)	1		
Multi	799 (43.9)	249 (31.2)	2.11	1.20–3.71	0.010*
Max PD T0					< 0.001***
4–5 mm	1382 (76.0)	134 (9.7)	1		
6 mm	293 (16.1)	136 (46.4)	7.11	4.19–12.1	< 0.001***
≥ 7 mm	143 (7.9)	105 (73.4)	25.9	10.6–63.4	< 0.001***
≥ 7 mm vs. 6 mm (ref.)			3.65	1.55–8.55	0.003**
Max Interprox. CAL T0	4.9 ± 1.4		0.96	0.81–1.14	0.653
Max RBL					0.004**
< 15%	432 (24.1)	53 (12.3)	1		
15%–33%	1085 (60.5)	209 (19.3)	1.56	0.84–2.91	0.160
> 33%	276 (15.4)	112 (40.6)	3.61	1.68–7.77	0.001**
> 33% vs. 15%–33% (ref.)			2.31	1.20–4.42	0.012*
Vertical defect > 3 mm					
No	1659 (92.5)	320 (19.3)	1		
Yes	135 (7.5)	55 (40.7)	1.28	0.62–2.67	0.503
BOP T0					
No	406 (23.3)	48 (11.8)	1		
Yes	1338 (76.7)	313 (23.4)	2.71	1.37–5.38	0.004**

Abbreviations: BoP, bleeding on probing; CAL, clinical attachment level; CI, confidence interval; OR, odds ratio; PD, pocket depth; RBL, radiographic bone loss; tIRR, tooth‐level incomplete resolution rate.

*p < 0.05, Wald test; ***p < 0.001, Wald test.

In current and former smokers, teeth showed a significantly higher tIRR rate than in non‐smokers (OR = 2.19, 95% CI: 1.01–4.78; *p* = 0.048 and OR = 2.02, 95% CI: 1.02–4.01; *p* = 0.044; respectively), while multi‐rooted teeth had a significantly higher tIRR than single‐rooted teeth (OR = 2.30, 95% CI: 1.37–3.88; *p* = 0.002).

At the same time, dental elements with the highest baseline site ≥ 7 mm are significantly more likely to exhibit the persistence of a PD > 4 mm at follow‐up compared to elements with a baseline site of 6 mm or 4–5 mm (OR = 3.14, 95% CI: 1.07–9.17, *p* = 0.036; OR = 16.9, 95% CI: 6.01–47.07, *p* < 0.001, respectively). Similarly, teeth associated with a PD of 6 mm at baseline show a significantly higher risk compared to teeth with a baseline PD of 4–5 mm (OR = 5.59, 95% CI: 3.60–8.69, *p* < 0.001). Conversely, baseline CAL and RBL did not show any significant association with tISR.

**FIGURE 2:**
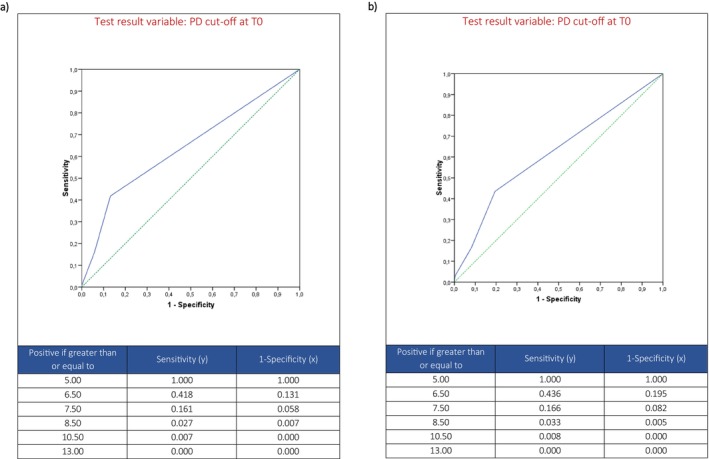
Receiver Operating Characteristic (ROC) curve estimating the optimal PD cutoff value for predicting tooth‐level incomplete success rate (tISR), based on the characteristics of (a) C1 and (b) C2. In both graphs, the vertex of the AUC polygon corresponds to a PD cutoff value of 6.5 mm, where Youden's Index (Sensitivity + 1–Specificity) reaches its maximum value. PD, pocket depth.

The ROC curve estimated at 6.5 mm the cutoff value of PD at T0, which implied a significantly greater probability of incomplete success at tooth‐level. The AUC rated the diagnostic accuracy at 64% (95% CI: 59%–70%) (Figure 2a).

#### 3.4.2 C2

In total, 20.6% of the treated teeth exhibited C2 at re‐evaluation (95% CI: 18.8%–22.5%), while 79.4% reported PD values ≤ 5 mm without BoP (95% CI: 77.5%–81.2%).

Univariate analysis of the association between independent tooth‐related variables and tISR is displayed in Table S4b. Statistically significant variables were then analysed with multi‐level binary logistic regression with GEE (Table 3b).

In current smokers, teeth showed a tendency toward a higher tIRR compared to non‐smokers (OR = 1.96, 95% CI: 0.90–4.27; *p* = 0.090), while multi‐rooted teeth exhibited a significantly higher tISR compared to single‐rooted teeth (OR = 2.11, 95% CI: 1.20–3.71; *p* = 0.01).

Additionally, dental elements with the highest baseline site ≥ 7 mm are significantly more likely to present C2 at follow‐up compared to those with a baseline site of 6 mm or 4–5 mm (OR = 3.65, 95% CI: 1.55–8.55, *p* = 0.003; OR = 25.9, 95% CI: 10.6–63.4, *p* < 0.001, respectively). Similarly, teeth with a baseline PD of 6 mm show a significantly higher risk compared to those with a baseline PD of 4–5 mm (OR = 7.11, 95% CI: 4.19–12.1, *p* < 0.001).

Finally, while baseline CAL does not affect the tISR, dental elements with the highest baseline site associated with an RBL > 33% are significantly more likely to exhibit C2 at follow‐up compared to elements with a baseline RBL between 15% and 33% or < 15% (OR = 2.31, 95% CI: 1.20–4.42, *p* = 0.012; OR = 3.61, 95% CI: 1.68–7.77, *p* = 0.001, respectively).

The ROC curve estimated at 6.5 mm the cutoff value of PD at T0 which implied a significantly greater probability of incomplete success at tooth‐level. The AUC rated the diagnostic accuracy at 62% (95% CI: 57%–67%) (Figure 2b).

## 4 Discussion

The aim of this study was to estimate the ISR of NSPT specifically performed by registered dental hygienists, in order to identify both patients and teeth requiring additional treatment, most likely surgical, and thus necessitating referral to a periodontist or a dentist specialised in periodontology. Primarily, the study sought to evaluate the impact of periodontitis staging and grading on the success of NSPT, while also assessing the influence of various systemic, clinical and radiographic factors. The ISR was evaluated at both the patient and tooth levels, with a dual analysis at each level that considered the persistence of C1 or C2 conditions. During data collection, selecting the pathological site with the highest PD value for each tooth was consistent with the statistical analysis conducted at the tooth level. Recording only the site with the highest PD for each tooth at baseline and at T1 allowed identification of how many and which teeth required further treatment as part of Step 3 of periodontal therapy. Even if a single tooth presented multiple sites with pathological probing depths on different surfaces, the surgical procedure would involve the entire tooth in the same session. This approach enabled an assessment of the likelihood that a tooth as a whole might require additional treatment when associated with specific baseline clinical or radiographic characteristics.

However, if the objective had been to determine how many sites required further instrumentation before surgical therapy, selecting only the site with the highest PD would have limited accurate quantification. This approach might have overestimated therapy effectiveness, as multiple pathological probing depths could remain on a single tooth. Furthermore, this analysis does not estimate the likelihood of a specific site with a given pocket depth achieving ‘closure’, as the sites selected at baseline and follow‐up (T1) could differ. Indeed, it is also important to note that the presence of C1 or C2 conditions at follow‐up may not depend on the initially selected site but rather on another site on the same tooth.

Since this study neither conducted a site‐level statistical analysis nor aimed to measure clinical attachment gain or probing depth reduction after NSPT, the adopted data collection methodology can be considered sufficiently reliable to determine the number of teeth and, consequently, the number of patients requiring additional treatment after NSPT and referral to a periodontist for surgical therapy.

Based on these considerations, the pISR was 75.9% and 65.4%, reflecting the proportion of patients with at least one site showing persistence of C1 or C2, respectively. Interestingly, when the pISR was evaluated based on C1, the stage of periodontitis emerged as the only factor significantly increasing the risk of not achieving complete success, with patients in Stage 3–4 having a higher pISR compared to those in Stage 1–2.

Indeed, the stage of periodontitis, which essentially defines the severity and complexity of the treatment required, serves as a significant predictor at the patient level for a higher ISR of treatment in our study. Additionally, as shown in Table S3, 59.2% of patients with Stage 3–4 periodontitis presented at least one defect with an infrabony component > 3 mm, representing an additional factor that complicates the success of NSPT. Conversely, in the tooth‐level analysis, the presence of an infrabony defect with a vertical component > 3 mm was significantly associated with tISR only in the univariate analysis, but not in the multivariable model. This finding is likely explained by the effect of PD in the multivariable model (a parameter not considered in the patient‐level analysis), which appears to outweigh the effect of the vertical defect itself, emerging as one of the main predictors of tISR

Conversely, no significance of grading on pISR was detected for both analyses. On the one hand, this result could be a consequence of the rigidity of the criterion that defined the complete/incomplete success of NSPT, as explained in the material and methods section. The factors covered in the grade (smoking, diabetes and disease progression) do not have such a strong influence as to override significance in the multiple models in the patient‐level analysis. In fact, the persistence of even a single site presenting C1 or C2 is extremely likely to be found even in patients free from factors that could complicate the response to periodontal therapy. Second, another very interesting aspect is the young age that characterises the sample of our study classified with a grade C, from which one would expect a worse response to therapy. In fact, 34.5% of patients included with grade C periodontitis are aged ≤ 35 years, compared to 8.2% and 9.6% of patients classified with grade B and grade A, respectively. From the conclusions of a study by Trombelli et al. patients younger than 35 years showed a significantly lower risk of having residual pockets following treatment compared to the older group [20]. In this way, the pISR of patients with grade C almost equivalent that of patients with grade B (82.8% vs. 78.1%, respectively in the C1‐based analysis; and 75.9% vs. 65.9%, respectively in the C2‐based analysis) after therapy could be explained.

A previous study aimed to evaluate the effects of disease severity defined by the staging and grading system on the periodontal response to therapy after 1 year. However, the results showed that the new classification system did not adequately reflect the periodontal response to therapy in the group of patients they analysed [16]. Other studies, however, have evaluated the effects of staging and grading at the time of diagnosis on other outcomes, such as tooth loss due to periodontal reasons, the need for additional surgical therapy during maintenance, and consequently, the costs of periodontal therapy [14, 15, 21, 22]. Interestingly, it emerged that the severity of the disease has a significant impact on all of these parameters, as patients with higher stages and grades are more likely to lose more teeth due to periodontal reasons, require additional surgical treatments during the maintenance phase, and therefore have to invest more financially compared to patients with less severe and less complex periodontal conditions.

The persistence of pathological sites, as defined by the criteria for C1 and C2, was observed in 28.6% and 20.6% of teeth, respectively. In both tooth‐level analyses, baseline pocket depth and the presence of multi‐rooted teeth were the characteristics most significantly associated with an increased risk of tISR. Furthermore, when using the persistence of C2 at the tooth level as a criterion for incomplete success, baseline RBL was also identified as a significant factor influencing the effectiveness of NSPT.

When the presence of C1 was used as the criterion for incomplete success, smokers and former smokers exhibited a significantly higher percentage of pathological sites compared to non‐smokers.

Several authors have adopted a PD threshold of 4 mm beyond which ‘incomplete pocket closure’ can be defined [5, 10, 11]. Specifically, the results of a multicenter randomised study by Tomasi et al. were consistent with our success rates, although these authors conducted a 6‐month follow‐up. In fact, their research showed that non‐surgical periodontal therapy resulted in approximately 70% of teeth achieving pocket ‘closure’ (i.e., residual PD ≤ 4 mm), slightly lower than the 71.4% attained by the dental hygienists in our current study, when considering the C1 criterion [10].

A study by Citterio et al., unlike our research, conducted an analysis at the site level [21]. However, using the same criteria associated with C1 and C2 from our study to define pocket ‘closure’, they reported percentages of sites with non‐pathological probing depths of 70.2% and 58.4%, respectively, after NSPT. While differences in the level of analysis prevent a direct comparison between this study and ours, Citterio et al.'s findings similarly reveal a discrepancy in the success rate of therapy depending on whether success is evaluated solely based on PD or using both PD and BoP. The proportion of teeth and sites that did not require further treatment after NSPT was notably lower when BoP was included in the evaluation.

Contrastingly, some authors have highlighted the requirement for additional therapy when pockets with depths > 3 or ≥ 6 mm persist [23, 24]. Undoubtedly, this discrepancy has hindered a direct comparison of our results with those of other studies.

The incomplete success rates of 75.9% and 65.4% at the patient level, based on the C1 and C2 criteria respectively, as determined in the present study, can be easily attributed to the stringent evaluation criteria applied. These criteria classified the therapy as ‘not fully successful’, even if only a single pocket with PD > 4 mm, a pocket with PD = 5 mm and the presence of BoP, or a pocket with PD ≥ 6 mm persisted at the patient level after periodontal reassessment at follow‐up.

This may also explain the lack of significant differences observed in the pISR between smokers and non‐smokers, as well as between diabetics and non‐diabetics in the multivariable patient‐level analyses. Moreover, the relatively small sample size, particularly for diabetic patients, could account for these findings.

The results of this analysis, however, should also be weighed against those from the tooth‐level analysis, which, consistent with existing literature, reports a considerably higher rate of teeth achieving the therapy endpoint [25]. Therefore, the conclusions of the present study must enthusiastically highlight the outcomes achievable with NSPT alone, despite the less favourable patient‐level results.

Clearly, the results highlighted that the probability of a tooth requiring additional treatment, likely surgical, after NSPT depends on the initial pocket depth, the tooth anatomy, the patient's smoking status (whether current or former smoker), and likely the complexity and severity of the disease. Keeping these factors in mind, clinicians can assess the state of the disease before treatment and define a tailored treatment approach based on the patient's specific needs, while setting clear and realistic treatment goals.

Drawing on the insights presented by Kwok and Caton during the formulation of a prognostic system for individual teeth, the present study deemed it appropriate to include systemic factors such as diabetes and smoking habits in the models utilised for tooth‐level analysis [26]. In particular, estimates from the models based on the analysis using C1 revealed robust significance, indicating that teeth belonging to smokers or former smokers had tISR approximately twice as high compared to teeth of non‐smokers. Our findings are in agreement with the study by Citterio et al., who, although not detecting a significant influence of smoking in the patient‐level analysis, reported a prevalence of sites ≤ 4 mm of 76.50% in non‐smokers and of 64.16% in smokers, demonstrating a significant correlation between the smoking status and pocket ‘closure’ at site level [21]. Similarly, a recent systematic review by Chang et al. clearly reported a less favourable response to non‐surgical periodontal therapy in smokers, with significantly less PD reduction [27].

In the present study, the multiple models in both analyses at tooth‐level showed that positive predictors of incomplete success included tooth type and PD at T0, while only the analysis based on C2 also considered RBL. These findings are not new, as they have already been extensively highlighted in previous studies [23, 24, 28]. Additionally, this report found that pockets at molars had an tISR more than double that of single‐rooted teeth. In fact, scaling and root planning procedures are more challenging in multi‐rooted teeth, due to the macroscopic and microscopic anatomical complexities of the furcation area and the poorer accessibility for subgingival instrumentation [29, 30].

The collection and retrospective analysis of the results and clinical data extracted from the medical records showed intrinsic limitations that characterised the present study. First, several uncalibrated operators with varying levels of experience and clinical skills participated in the study, as it was conducted in an academic setting. Second, different therapeutic protocols tailored to the patients' needs may have been employed. Nonetheless, it is highly likely that all hygienists adhered to traditional non‐surgical periodontal therapy methods, including ultrasonic and hand instrumentation, combined with oral hygiene instructions. Third, smoking status and diabetes were not assessed through objective measures but were instead self‐reported by the patients.

Lastly, another limitation was the considerable variability in the re‐evaluation time. A shorter re‐evaluation period, especially for sites with greater pocket depths, may not have been sufficient to fully capitalise on the healing process, leading to an overestimation of pathological sites following non‐surgical periodontal therapy.

Prospective randomised‐controlled studies should be conducted to confirm which factors may hinder a complete success of NSPT by the dental hygienist, to have more robust implications especially for clinical practice.

## 5 Conclusion

The staging of periodontitis emerged as the primary patient‐related factor significantly influencing the outcomes of NSPT performed by the hygienist, with teeth considered as having incomplete success if a PD > 4 mm (C1) persisted at follow‐up. Despite a relatively low tISR confirming NSPT effectiveness, baseline PD, multi‐rooted tooth anatomy, and current or past smoking habits were associated with higher risk of teeth retaining pathological sites, potentially requiring additional treatment.

## 6 Clinical Relevance

### 6.1 Scientific Rationale for the Study

NSPT is effective in stabilising periodontitis; however, disease complexity and tooth factors can impact outcomes. This study assessed the influence of periodontitis staging and grading, along with clinical parameters, on the rate of incomplete success in non‐surgical therapy specifically performed by dental hygienists.

### 6.2 Principal Findings

Periodontitis staging, smoking habits, tooth type and baseline values of PD and RBL play a crucial role in influencing the success of NSPT.

### 6.3 Practical Implications

Periodontitis staging, smoking, molar involvement and probing depth > 6.5 mm can hinder NSPT success. Dental professionals must consider these factors to set achievable goals from diagnosis and tailor therapy to the patient's needs and expectations.

## Author Contributions

Study conception and design: Andrea Ravidà, Matteo Serroni and Muhammad H.A. Saleh. Data collection: Obada Mandil, Chiara Galano, Aml Abukraa and Aliyah Alsubhi. Analysis of data: Andrea Ravidà and Muhammad H.A. Saleh. Interpretation of data: Andrea Ravidà, Matteo Serroni, Danielle Rulli and Muhammad H.A. Saleh. Article writing and editing: Andrea Ravidà and Matteo Serroni. Critical revising and editing: Danielle Rulli and Muhammad H.A. Saleh.

## Funding

The authors have nothing to report.

## Conflicts of Interest

The authors declare no conflicts of interest.

## Supporting information


**Table S1:** Incomplete success rate (pISR) based on patient‐related clinical variables, assessed according to (a) C1 and (b) C2. Results of simple binary logistic regression using GEE.

**Table S2:** pISR in Stage III‐IV patients with vertical defect (with infrabony component > 3 mm), assessed according to (a) C1 and (b) C2. Results of multiple binary logistic regression.

**Table S3:** pISR in Stage III‐IV patients with PD ≥ 7 mm, assessed according to (a) C1 and (b) C2. Results of multiple binary logistic regression.

**Table S4:** Results of simple binary logistic regression using GEE on the sample of teeth with pathological PD (≥ 4 mm) at baseline. (a) Incomplete success rate (tISR) assessed based on tooth‐related clinical variables influencing the presence of C1. (b) Incomplete success rate (tISR) assessed based on tooth‐related clinical variables influencing the presence of C2.

## Data Availability

The data that support the findings of this study are available from the corresponding author upon reasonable request.
