# Supplementary material for: Influence of Staging and Grading and Multiple Factors on the Success of Non‐Surgical Periodontal Therapy Performed by Dental Hygienists: A Retrospective Analysis
Source: Int J Dent Hyg. 2026 Feb 22;24(3):369–81. doi: 10.1111/idh.70036 (PMC13309215; doi:10.1111/idh.70036)
Supplement: Supplementary file 1 — Table S1: Incomplete success rate (pISR) based on patient‐related clinical variables, assessed according to (a) C1 and (b) C2. Results of simple binary logistic regression using GEE. [file IDH-24-369-s002.docx]

**Supplementary Table 1.** Incomplete success rate (pISR) based on patient-related clinical variables, assessed according to a) C1 and b) C2. Results of simple binary logistic regression using GEE.

**a)**

|  | **Total** |  | **INCOMPLETE SUCCESS rarte (pISR)** | **OR** | **95%CI** | **p-value** |
| --- | --- | --- | --- | --- | --- | --- |
| **N of patients** | 133 |  | 101 (75.9%) |  |  |  |
| **AGE (years)** | 52.8 ± 15.4 |  |  | 0.99 | 0.97 – 1.02 | 0.486 |
| **SEX** |  |  |  |  |  |  |
| Male | 72 (54.1%) |  | 58 (80.6%) | 1 |  |  |
| Female | 61 (45.9%) |  | 43 (70.5%) | 0.58 | 0.26 – 1.29 | 0.179 |
| **SMOKING** |  |  |  |  |  | 0.228 |
| No | 87 (65.4%) |  | 62 (71.3%) | 1 |  |  |
| Former | 22 (16.5%) |  | 19 (86.4%) | 2.55 | 0.69 – 9.40 | 0.159 |
| Current | 24 (18.0%) |  | 20 (83.3%) | 2.02 | 0.63 – 6.49 | 0.240 |
| **DIABETES** |  |  |  |  |  |  |
| No | 116 (87.9%) |  | 84 (72.4%) | 1 |  |  |
| Yes | 16 (12.1%) |  | 16 (100.0%) | 5.71 | 0.73 – 45.0 | 0.098 |
| **STAGE** |  |  |  |  |  |  |
| 2 | 62 (46.6%) |  | 42 (67.7%) | 1 |  |  |
| 3-4 | 71 (53.4%) |  | 59 (83.1%) | 2.34 | 1.03 – 5.30 | **0.041*** |
| **GRADE** |  |  |  |  |  | 0.218 |
| A | 31 (23.3%) |  | 20 (64.5%) | 1 |  |  |
| B | 73 (54.9%) |  | 57 (78.1%) | 1.96 | 0.78 – 4.92 | 0.152 |
| C | 29 (21.8%) |  | 24 (82.8%) | 2.64 | 0.79 – 8.87 | 0.117 |
| **GRADE in STAGE II** |  |  |  |  |  | 0.653 |
| 2A | 29 (46.8%) |  | 18 (62.1%) | 1 |  |  |
| 2B | 30 (48.4%) |  | 22 (73.3%) | 1.68 | 0.56 – 5.07 | 0.356 |
| 2C | 3 (4.8%) |  | 2 (66.7%) | 1.22 | 0.10 – 15.1 | 0.876 |
| **GRADE in STAGE III** |  |  |  |  |  |  |
| 3A | 2 (2.8%) |  | 2 (100.0%) | -- |  |  |
| 3B | 43 (60.6%) |  | 35 (81.4%) | 1 |  |  |
| 3C | 26 (36.6%) |  | 22 (84.6%) | 1.26 | 0.34 – 4.68 | 0.733 |
| **EXTENT** |  |  |  |  |  |  |
| Localized | 55 (41.4%) |  | 42 (76.4%) | 1 |  |  |
| Generalized | 78 (58.6%) |  | 59 (75.6%) | 0.96 | 0.43 – 2.16 | 0.924 |

pISR, patient-level incomplete success rate; OR, odds ratio; CI, confidence interval.

* p<0.05, Wald test

**b)**

|  | **Total** |  | **INCOMPLETESUCCESS rate (pISR)** | **OR** | **95%CI** | **p-value** |
| --- | --- | --- | --- | --- | --- | --- |
| **N of patients** | 133 |  | 87 (65.4) |  |  |  |
| **AGE (years)** | 52.8 ± 15.4 |  |  | 1.00 | 0.98 – 1.02 | 0.976 |
| **SEX** |  |  |  |  |  |  |
| Male | 72 (54.1) |  | 49 (68.1) | 1 |  |  |
| Female | 61 (45.9) |  | 38 (62.3) | 0.78 | 0..38 – 1.59 | 0.487 |
| **SMOKING** |  |  |  |  |  | 0.482 |
| No | 87 (65.4) |  | 54 (62.1) | 1 |  |  |
| Former | 22 (16.5) |  | 15 (68.2) | 1.31 | 0.48 – 3.55 | 0.596 |
| Current | 24 (18.0) |  | 18 (75.0) | 1.83 | 0.66 – 5.09 | 0.244 |
| **DIABETES** |  |  |  |  |  |  |
| No | 116 (87.9) |  | 72 (62.1) | 1 |  |  |
| Yes | 16 (12.1) |  | 14 (87.5) | 4.28 | 0.93 – 19.7 | 0.062 |
| **STAGE** |  |  |  |  |  |  |
| 2 | 62 (46.6) |  | 33 (53.2) | 1 |  |  |
| 3-4 | 71 (53.4) |  | 54 (76.1) | 2.79 | 1.33 – 5.84 | **0.006**** |
| **GRADE** |  |  |  |  |  | **0.026*** |
| A | 31 (23.3) |  | 14 (45.2) | 1 |  |  |
| B | 73 (54.9) |  | 51 (69.9) | 2.82 | 1.18 – 6.69 | **0.019*** |
| C | 29 (21.8) |  | 22 (75.9) | 3.82 | 1.26 – 11.5 | **0.018*** |
| C vs. B (ref.) |  |  |  | 1.36 | 0.51 – 3.64 | 0.545 |
| **GRADE in STAGE 2** |  |  |  |  |  | 0.455 |
| 2A | 29 (46.8) |  | 13 (44.8) | 1 |  |  |
| 2B | 30 (48.4) |  | 18 (60.0) | 1.85 | 0.66 – 5.19 | 0.246 |
| 2C | 3 (4.8) |  | 2 (66.7) | 2.46 | 0.20 – 30.3 | 0.482 |
| **GRADE in STAGE 3** |  |  |  |  |  |  |
| 3A | 2 (2.8) |  | 1 (50) | -- |  |  |
| 3B | 43 (60.6) |  | 33 (76.7) | 1 |  |  |
| 3C | 26 (36.6) |  | 20 (76.9) | 1.01 | 0.32 – 3.20 | 0.986 |
| **EXTENT** |  |  |  |  |  |  |
| Localized | 55 (41.4) |  | 35 (63.6) | 1 |  |  |
| Generalized | 78 (58.6) |  | 52 (66.7) | 1.14 | 0.55 – 2.36 | 0.718 |

pISR, patient-level incomplete success rate; OR, odds ratio; CI, confidence interval.

* p<0.05, Wald test
