# Supplementary material for: Influence of Staging and Grading and Multiple Factors on the Success of Non‐Surgical Periodontal Therapy Performed by Dental Hygienists: A Retrospective Analysis
Source: Int J Dent Hyg. 2026 Feb 22;24(3):369–81. doi: 10.1111/idh.70036 (PMC13309215; doi:10.1111/idh.70036)
Supplement: Supplementary file 2 — Table S2: pISR in Stage III‐IV patients with vertical defect (with infrabony component > 3 mm), assessed according to (a) C1 and (b) C2. Results of multiple binary logistic regression. [file IDH-24-369-s004.docx]

**Supplementary Table 2**. pISR in Stage III-IV patients with vertical defect (with infrabony component >3mm), assessed according to a) C1 and b) C2**.** Results of multiple binary logistic regression.

**a)**

|  | **Total** |  | **INCOMPLETESUCCESS rate (pISR)** | **OR** | **95%CI** | **p-value** |
| --- | --- | --- | --- | --- | --- | --- |
| **N of patients** | 71 |  | 59 (83.1) |  |  |  |
| **VERTICAL DEFECTS >3mm** |  |  |  |  |  |  |
| No | 29 (40.8) |  | 23 (79.3) | 1 |  |  |
| Yes | 42 (59.2) |  | 36 (85.7) | 2.77 | 0.59 – 12.9 | 0.195 |
| **AGE (years)** |  |  |  | 0.94 | 0.90 – 0.99 | **0.029*** |
| **SEX** |  |  |  |  |  |  |
| Male | 41 (57.7) |  | 36 (87.8%) | 1 |  |  |
| Female | 30 (42.3) |  | 23 (76.7%) | 0.65 | 0.16 – 2.61 | 0.547 |
| **SMOKING** |  |  |  |  |  | 0.953 |
| No | 43 (60.6) |  | 35 (81.4) | 1 |  |  |
| Former | 12 (16.9) |  | 10 (83.3) | 1.15 | 0.17 – 7.62 | 0.883 |
| Current | 16 (22.5) |  | 14 (87.5) | 0.80 | 0.12 – 5.63 | 0.826 |
| **DIABETES** |  |  |  |  |  |  |
| No | 59 (83.1) |  | 47 (79.7) | 1 |  |  |
| Yes | 12 (16.9) |  | 12 (100) | -- | -- | -- |

pISR, patient-level incomplete success rate; OR, odds ratio; CI, confidence interval.

**b)**

|  | **Total** |  | **INCOMPLETESUCCESS rate (pISR)** | **OR** | **95%CI** | **p-value** |
| --- | --- | --- | --- | --- | --- | --- |
| **N of patients** | 71 |  | 54 (76.1) |  |  |  |
| **VERTICAL DEFECTS >3mm** |  |  |  |  |  |  |
| No | 29 (40.8) |  | 19 (65.5) | 1 |  |  |
| Yes | 42 (59.2) |  | 35 (83.3) | 3.97 | 1.04 – 15.1 | **0.043*** |
| **AGE (years)** |  |  |  | 0.95 | 0.90 – 0.99 | **0.018*** |
| **SEX** |  |  |  |  |  |  |
| Male | 41 (57.7) |  | 32 (78.0%) | 1 |  |  |
| Female | 30 (42.3) |  | 22 (73.3%) | 0.89 | 0.25 – 3.13 | 0.850 |
| **SMOKING** |  |  |  |  |  | 0.979 |
| No | 43 (60.6) |  | 32 (74.4) | 1 |  |  |
| Former | 12 (16.9) |  | 9 (75.0) | 1.18 | 0.23 – 6.08 | 0.846 |
| Current | 16 (22.5) |  | 13 (81.3) | 0.99 | 0.19 – 5.24 | 0.998 |
| **DIABETES** |  |  |  |  |  |  |
| No | 59 (83.1) |  | 44 (74.6) | 1 |  |  |
| Yes | 12 (16.9) |  | 10 (83.3) | 2.70 | 0.34 – 21.3 | 0.347 |

pISR, patient-level incomplete success rate; OR, odds ratio; CI, confidence interval.
