# Supplementary material for: Influence of Staging and Grading and Multiple Factors on the Success of Non‐Surgical Periodontal Therapy Performed by Dental Hygienists: A Retrospective Analysis
Source: Int J Dent Hyg. 2026 Feb 22;24(3):369–81. doi: 10.1111/idh.70036 (PMC13309215; doi:10.1111/idh.70036)
Supplement: Supplementary file 3 — Table S3: pISR in Stage III‐IV patients with PD ≥ 7 mm, assessed according to (a) C1 and (b) C2. Results of multiple binary logistic regression. [file IDH-24-369-s003.docx]

**Supplementary Table 3**. pISR in Stage III-IV patients with PD ≥7mm, assessed according to a) C1 and b) C2**.** Results of multiple binary logistic regression.

**a)**

|  | **Total** |  | **INCOMPLETE SUCCESS**  **rate (pISR)** | **OR** | **95%CI** | **p-value** |
| --- | --- | --- | --- | --- | --- | --- |
| **N of patients** | 71 |  | 59 (83.1) |  |  |  |
| **POCKETS >=7mm** |  |  |  |  |  |  |
| No | 32 (45.1) |  | 22 (68.8) | 1 |  |  |
| Yes | 39 (54.9) |  | 37 (94.9) | 10.8 | 4.19 – 146.5 | **<0.001***** |
| **AGE (years)** |  |  |  | 0.94 | 0.88 – 0.99 | **0.032*** |
| **SEX** |  |  |  |  |  |  |
| Male | 41 (57.7) |  | 36 (87.8%) | 1 |  |  |
| Female | 30 (42.3) |  | 23 (76.7%) | 0.75 | 0.17 – 3.31 | 0.703 |
| **SMOKING** |  |  |  |  |  | 0.928 |
| No | 43 (60.6) |  | 35 (81.4) | 1 |  |  |
| Former | 12 (16.9) |  | 10 (83.3) | 1.19 | 0.16 – 8.72 | 0.861 |
| Current | 16 (22.5) |  | 14 (87.5) | 0.74 | 0.10 – 5.48 | 0.735 |
| **DIABETES** |  |  |  |  |  |  |
| No | 59 (83.1) |  | 47 (79.7) | 1 |  |  |
| Yes | 12 (16.9) |  | 12 (100) | -- | -- | -- |

pISR, patient-level incomplete success rate; OR, odds ratio; CI, confidence interval.

** p<0.01, Wald test

**b)**

|  | **Total** |  | **INCOMPLETESUCCESS rate (pISR)** | **OR** | **95%CI** | **p-value** |
| --- | --- | --- | --- | --- | --- | --- |
| **N of patients** | 71 |  | 54 (76.1) |  |  |  |
| **POCKETS >=7mm** |  |  |  |  |  |  |
| No | 32 (45.1) |  | 17 (53.1) | 1 |  |  |
| Yes | 39 (54.9) |  | 37 (94.9) | 24.8 | 4.19 – 146.5 | **<0.001***** |
| **AGE (years)** |  |  |  | 0.93 | 0.88 – 0.99 | **0.016*** |
| **SEX** |  |  |  |  |  |  |
| Male | 41 (57.7) |  | 32 (78.0%) | 1 |  |  |
| Female | 30 (42.3) |  | 22 (73.3%) | 1.21 | 0.29 – 5.03 | 0.791 |
| **SMOKING** |  |  |  |  |  | 0.893 |
| No | 43 (60.6) |  | 32 (74.4) | 1 |  |  |
| Former | 12 (16.9) |  | 9 (75.0) | 1.46 | 0.23 – 9.33 | 0.689 |
| Current | 16 (22.5) |  | 13 (81.3) | 1.43 | 0.21 – 9.60 | 0.710 |
| **DIABETES** |  |  |  |  |  |  |
| No | 59 (83.1) |  | 44 (74.6) | 1 |  |  |
| Yes | 12 (16.9) |  | 10 (83.3) | 1.16 | 0.12 – 11.0 | 0.896 |

pISR, patient-level incomplete success rate; OR, odds ratio; CI, confidence interval.

** p<0.01, Wald test
