# Supplementary material for: Influence of Staging and Grading and Multiple Factors on the Success of Non‐Surgical Periodontal Therapy Performed by Dental Hygienists: A Retrospective Analysis
Source: Int J Dent Hyg. 2026 Feb 22;24(3):369–81. doi: 10.1111/idh.70036 (PMC13309215; doi:10.1111/idh.70036)
Supplement: Supplementary file 4 — Table S4: Results of simple binary logistic regression using GEE on the sample of teeth with pathological PD (≥ 4 mm) at baseline. (a) Incomplete success rate (tISR) assessed based on tooth‐related clinical variables influencing the presence of C1. (b) Incomplete success rate (tISR) assessed based on tooth‐related clinical variables influencing the presence of C2. [file IDH-24-369-s001.docx]

**Supplementary Table 4**. Results of simple binary logistic regression using GEE on the sample of teeth with pathological PD (≥4mm) at baseline. a) Incomplete success rate (tISR) assessed based on tooth-related clinical variables influencing the presence of C1. b) Incomplete success rate (tISR) assessed based on tooth-related clinical variables influencing the presence of C2.

**a)**

|  | **Total** |  | **INCOMPLETESUCCESS rate (tISR)** | **OR** | **95%CI** | **p-value** |
| --- | --- | --- | --- | --- | --- | --- |
| **N of teeth** | 1,818 |  | 520 (28.6) |  |  |  |
| **AGE (years)** | 49.1 ± 15.4 |  |  | 1.01 | 0.99 – 1.02 | 0.214 |
| **SEX** |  |  |  |  |  |  |
| Male | 1,053 (57.9) |  | 311 (29.5) | 1 |  |  |
| Female | 765 (42.1) |  | 209 (27.3) | 0.90 | 0.55 – 1.46 | 0.660 |
| **SMOKING** |  |  |  |  |  | **0.022*** |
| No | 1,214 (66.8) |  | 294 (24.2) | 1 |  |  |
| Former | 272 (15.0) |  | 93 (34.2) | 1.63 | 0.92 – 2.87 | 0.094 |
| Current | 332 (18.3) |  | 133 (40.1) | 2.09 | 1.21 – 3.62 | **0.008**** |
| Current vs. Former (ref.) |  |  |  | 1.29 | 0.68 – 2.45 | 0.444 |
| **DIABETES** |  |  |  |  |  |  |
| No | 1,578 (87.2) |  | 417 (26.4) | 1 |  |  |
| Yes | 232 (12.8) |  | 100 (43.1) | 2.11 | 1.08 – 4.11 | **0.029*** |
| **TOOTH TYPE** |  |  |  |  |  | **<0.001***** |
| I | 360 (19.8) |  | 61 (16.9) | 1 |  |  |
| C | 226 (12.4) |  | 34 (15.0) | 0.87 | 0.53 – 1.42 | 0.573 |
| PM | 569 (31.3) |  | 124 (21.8) | 1.37 | 0.90 – 2.08 | 0.148 |
| M | 663 (36.5) |  | 301 (45.4) | 4.08 | 2.54 – 6.54 | **<0.001***** |
| **MOLAR** |  |  |  |  |  |  |
| No | 1,155 (63.5) |  | 219 (19.0) | 1 |  |  |
| Yes | 663 (36.5) |  | 301 (45.4) | 3.55 | 2.66 – 4.76 | **<0.001***** |
| **ROOT TYPE** |  |  |  |  |  |  |
| Single | 1,019 (56.1) |  | 181 (17.8) | 1 |  |  |
| Multi | 799 (43.9) |  | 339 (42.4) | 3.41 | 2.58 – 4.51 | **<0.001***** |
| **ARCH** |  |  |  |  |  |  |
| Maxilla | 937 (51.5) |  | 283 (30.2) | 1 |  |  |
| Mandible | 881 (48.5) |  | 237 (26.9) | 0.85 | 0.65 – 1.11 | 0.229 |
| **Mean PD at T0** | 5.0 ± 1.0 |  |  | 4.04 | 3.17 – 5.16 | **<0.001***** |
| **PD at T0** |  |  |  |  |  | **<0.001***** |
| 4-5 mm | 1,382 (76.0) |  | 221 (16.0) | 1 |  |  |
| 6 mm | 293 (16.1) |  | 174 (59.4) | 7.68 | 5.32 – 11.1 | **<0.001***** |
| >=7 mm | 143 (7.9) |  | 125 (87.4) | 36.5 | 17.1 – 78.0 | **<0.001***** |
| >=7 mm vs. 6 mm (ref.) |  |  |  | 4.75 | 2.28 – 9.88 | **<0.001***** |
| **Mean CAL at T0** | 4.9 ± 1.4 |  |  | 1.51 | 1.29 – 1.76 | **<0.001***** |
| **RBL at T0** |  |  |  |  |  | **<0.001***** |
| <15% | 432 (24.1) |  | 87 (20.1) | 1 |  |  |
| 15-33% | 1,085 (60.5) |  | 295 (27.2) | 1.48 | 1.02 – 2.15 | **0.038*** |
| >33% | 276 (15.4) |  | 137 (49.6) | 3.91 | 2.28 – 6.69 | **<0.001***** |
| >33% vs. 15-33% (ref.) |  |  |  | 2.64 | 1.70 – 4.10 | **<0.001***** |
| **VERTICAL DEFECT>3mm** |  |  |  |  |  |  |
| No | 1,659 (92.5) |  | 451 (27.2) | 1 |  |  |
| Yes | 135 (7.5) |  | 69 (51.1) | 2.80 | 1.95 – 4.01 | **<0.001***** |
| **BOP at T0** |  |  |  |  |  |  |
| No | 406 (23.3) |  | 87 (21.4) | 1 |  |  |
| Yes | 1,338 (76.7) |  | 413 (30.9) | 1.64 | 1.08 – 2.48 | **0.020*** |
| **PROSTHESIS** |  |  |  |  |  |  |
| No | 1,614 (89.4) |  | 444 (27.5) | 1 |  |  |
| Yes | 191 (10.6) |  | 76 (39.8) | 1.74 | 1.15 – 2.64 | **0.009**** |
| **INTERPROX. RESTORATION** |  |  |  |  |  |  |
| No | 1,604 (88.8) |  | 451 (28.1) | 1 |  |  |
| Yes | 202 (11.2) |  | 69 (34.2) | 1.33 | 0.85 – 2.08 | 0.216 |
| **FURCATION** | N=663 non-missing |  |  |  |  |  |
| No | 434 (65.5) |  | 190 (43.8) | 1 |  |  |
| Yes | 229 (34.5) |  | 111 (48.5) | 1.21 | 0.76 – 1.93 | 0.427 |
| **DEGREE OF FURCATION** | N=229 furcation |  |  |  |  |  |
| Grade 1 | 184 (80.3) |  | 88 (47.8) | 1 |  |  |
| Grade 2 | 42 (18.3) |  | 22 (52.4) | 1.14 | 0.45 – 2.92 | 0.784 |
| Grade 3 | 3 (1.3) |  | 1 (33.3) |  |  |  |

tISR, tooth-level incomplete success rate; OR, odds ratio; CI, confidence interval; I, incisor; C, canine; PM, premolar; M, molar; PD, pocket depth; CAL, clinical attachment loss; RBL, radiographic bone loss; BoP, bleeding on probing.

* p<0.05, Wald test

** p<0.01, Wald test

*** p<0.001, Wald test

**b)**

|  | **Total** |  | **INCOMPLETESUCCESS rate (tISR)** | **OR** | **95%CI** | **p-value** |
| --- | --- | --- | --- | --- | --- | --- |
| **N of teeth** | 1,818 |  | 375 (20.6) |  |  |  |
| **AGE (years)** | 49.1 ± 15.4 |  |  | 1.01 | 0.99 – 1.03 | 0.195 |
| **SEX** |  |  |  |  |  |  |
| Male | 1,053 (57.9) |  | 217 (20.6) | 1 |  |  |
| Female | 765 (42.1) |  | 158 (20.7) | 1.00 | 0.58 – 1.74 | 0.992 |
| **SMOKING** |  |  |  |  |  | 0.161 |
| No | 1,214 (66.8) |  | 217 (17.9) | 1 |  |  |
| Former | 272 (15.0) |  | 65 (23.9) | 1.44 | 0.73 – 2.85 | 0.291 |
| Current | 332 (18.3) |  | 93 (28.0) | 1.79 | 0.97 – 3.31 | 0.065 |
| Current vs. Former (ref.) |  |  |  | 1.24 | 0.58 – 2.64 | 0.578 |
| **DIABETES** |  |  |  |  |  |  |
| No | 1,578 (87.2) |  | 296 (18.8) | 1 |  |  |
| Yes | 232 (12.8) |  | 77 (33.2) | 2.15 | 0.98 – 4.75 | 0.058 |
| **TOOTH TYPE** |  |  |  |  |  | **<0.001***** |
| I | 360 (19.8) |  | 45 (12.5) | 1 |  |  |
| C | 226 (12.4) |  | 23 (10.2) | 0.79 | 0.47 – 1.34 | 0.386 |
| PM | 569 (31.3) |  | 86 (15.1) | 1.25 | 0.79 – 1.96 | 0.340 |
| M | 663 (36.5) |  | 221 (33.3) | 3.50 | 2.13 – 5.76 | **<0.001***** |
| **MOLAR** |  |  |  |  |  | **<0.001***** |
| No | 1,155 (63.5) |  | 154 (13.3) | 1 |  |  |
| Yes | 663 (36.5) |  | 221 (33.3) | 3.25 | 2.30 – 4.59 | **<0.001***** |
| **ROOT TYPE** |  |  |  |  |  |  |
| Single | 1,019 (56.1) |  | 126 (12.4) | 1 |  |  |
| Multi | 799 (43.9) |  | 249 (31.2) | 3.21 | 2.35 – 4.38 | **<0.001***** |
| **ARCH** |  |  |  |  |  |  |
| Maxilla | 937 (51.5) |  | 202 (21.6) | 1 |  |  |
| Mandible | 881 (48.5) |  | 173 (19.6) | 0.89 | 0.67 – 1.18 | 0.889 |
| **Mean PD at T0** | 5.0 ± 1.0 |  |  | 3.62 | 2.69 – 4.86 | **<0.001***** |
| **PD at T0** |  |  |  |  |  | **<0.001***** |
| 4-5 mm | 1,382 (76.0) |  | 134 (9.7) | 1 |  |  |
| 6 mm | 293 (16.1) |  | 136 (46.4) | 8.07 | 5.44 – 11.9 | **<0.001***** |
| >=7 mm | 143 (7.9) |  | 105 (73.4) | 25.7 | 12.5 – 53.2 | **<0.001***** |
| >=7 mm vs. 6 mm (ref.) |  |  |  | 3.19 | 1.61 – 6.32 | **0.001**** |
| **Mean CAL at T0** | 4.9 ± 1.4 |  |  | 1.44 | 1.21 – 1.73 | **<0.001***** |
| **RBL at T0** |  |  |  |  |  | **<0.001***** |
| <15% | 432 (24.1) |  | 53 (12.3) | 1 |  |  |
| 15-33% | 1,085 (60.5) |  | 209 (19.3) | 1.71 | 1.13 – 2.58 | **0.011*** |
| >33% | 276 (15.4) |  | 112 (40.6) | 4.88 | 2.83 – 8.42 | **<0.001***** |
| >33% vs. 15-33% (ref.) |  |  |  | 2.86 | 1.84 – 4.47 | **<0.001***** |
| **VERTICAL DEFECT>3mm** |  |  |  |  |  |  |
| No | 1,659 (92.5) |  | 320 (19.3) | 1 |  |  |
| Yes | 135 (7.5) |  | 55 (40.7) | 2.88 | 1.93 – 4.29 | **<0.001***** |
| **BOP at T0** |  |  |  |  |  |  |
| No | 406 (23.3) |  | 48 (11.8) | 1 |  |  |
| Yes | 1,338 (76.7) |  | 313 (23.4) | 2.28 | 1.26 – 4.13 | **0.007**** |
| **PROSTHESIS** |  |  |  |  |  |  |
| No | 1,614 (89.4) |  | 322 (20.0) | 1 |  |  |
| Yes | 191 (10.6) |  | 53 (27.7) | 1.54 | 0.96 – 2.47 | 0.072 |
| **INTERPROX. RESTORATION** |  |  |  |  |  |  |
| No | 1,604 (88.8) |  | 318 (19.8) | 1 |  |  |
| Yes | 202 (11.2) |  | 57 (28.2) | 1.59 | 0.95 – 2.68 | 0.081 |
| **FURCATION** | N=663 non-missing |  |  |  |  |  |
| No | 434 (65.5) |  | 130 (30.0) | 1 |  |  |
| Yes | 229 (34.5) |  | 91 (39.7) | 1.54 | 0.90 – 2.64 | 0.114 |
| **DEGREE OF FURCATION** | N=229 furcation |  |  |  |  |  |
| Grade 1 | 184 (80.3) |  | 71 (38.6) | 1 |  |  |
| Grade 2 | 42 (18.3) |  | 19 (45.2) | 1.27 | 0.50 – 3.26 | 0.615 |
| Grade 3 | 3 (1.3) |  | 1 (33.3) |  |  |  |

tISR, tooth-level incomplete success rate; OR, odds ratio; CI, confidence interval; I, incisor; C, canine; PM, premolar; M, molar; PD, pocket depth; CAL, clinical attachment loss; RBL, radiographic bone loss; BoP, bleeding on probing.

* p<0.05, Wald test

** p<0.01, Wald test

*** p<0.001, Wald test
